# Gás Ectópico: Um Sinal de Potencial Infecção de Endoprótese Aórtica Antes do Surgimento de Sintomas

**DOI:** 10.36660/abc.20250591

**Published:** 2026-06-16

**Authors:** Hongliang Li, Ying Zhao, Qingyu Ji

**Affiliations:** 1 Second Affiliated Hospital of Baotou Medical College Department of radiology Baotou Inner Mongolia China Second Affiliated Hospital of Baotou Medical College – Department of radiology, Baotou, Inner Mongolia – China

**Keywords:** Angiografia por Tomografia Computadorizada, Correção Endovascular de Aneurisma, Procedimentos Endovasculares, Infecções

## Introdução

A infecção da endoprótese da aorta abdominal (EAA) é a complicação mais temida do reparo endovascular da aorta (EVAR) para aneurisma da aorta abdominal (AAA), com taxas de mortalidade relatadas de até 51,8%.^1^ Metanálises recentes enfatizam que, apesar dos avanços na técnica cirúrgica, o prognóstico da infecção da EAA permanece ruim devido ao diagnóstico tardio e à complexidade da explantação da endoprótese.^1,2^ O manejo da infecção da EAA é significativamente dificultado pela latência diagnóstica. Atualmente, a angiotomografia computadorizada (angioTC) é geralmente reservada para avaliação somente após o início dos sintomas infecciosos. Embora o exsudato periprotético e o gás ectópico sejam considerados características radiográficas marcantes, esses sinais são frequentemente sutis e diminutos durante os estágios iniciais, pré-sintomáticos, da infecção, tornando-os facilmente negligenciados. Apresentamos um caso em que um radiologista detectou gás ectópico periprotético diminuto por meio de tomografia computadorizada de alta resolução durante uma angioTC de rotina de acompanhamento, cinco dias antes do início dos sintomas infecciosos, alertando assim os médicos para a possibilidade de infecção da EAA. Com base neste caso, destacamos o valor crítico da tomografia computadorizada de alta resolução na identificação precoce de gás ectópico. Além disso, pretendemos demonstrar como o pós-processamento avançado — especificamente a renderização de volume (RV) e a renderização hiper-realista (RHR) — aprimora a visualização da infecção da EAA, permitindo assim o monitoramento longitudinal da progressão da infecção.

## Apresentação do caso

**Figura 1:**
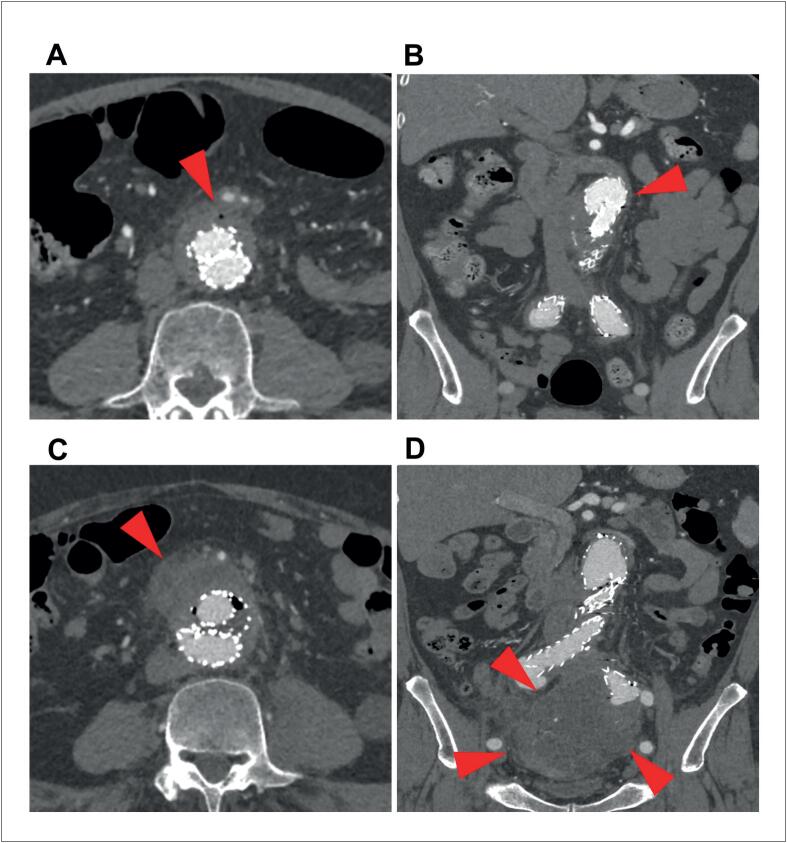
Achados de angiotomografia computadorizada angioTC antes e depois do início dos sintomas. (A) Imagem axial de angioTC de cortes finos (0,625 mm) obtida em consulta de rotina revela gás ectópico (seta vermelha) ao redor da endoprótese aórtica. (B) Reconstrução coronal do mesmo exame mostra leve espessamento da parede do vaso adjacente (seta). (C) Imagem axial de angioTC adquirida após o início dos sintomas demonstra uma massa de tecido mole (seta vermelha), representando uma coleção inflamatória, ao redor da prótese. (D) Reconstrução coronal localiza o abscesso periprotético resultante (seta vermelha) na cavidade pélvica.

**Figura 2:**
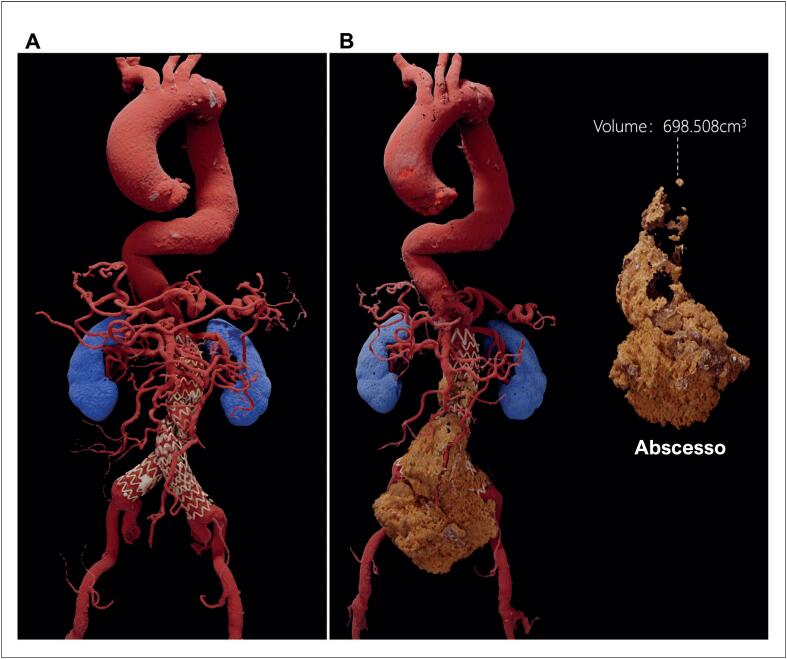
Reconstrução hiper-realista (RHR) por tomografia computadorizada antes e depois do início dos sintomas. (A) No acompanhamento de rotina, a RHR por TC não mostra deformação evidente da endoprótese nem alterações inflamatórias nos tecidos circundantes. (B) Após o desenvolvimento dos sintomas, a RHR por TC revela uma extensa coleção inflamatória (abscesso) ao redor da prótese.

Um homem de 78 anos, com histórico de EVAR para AAA dois anos antes, foi encaminhado para angioTC como parte de seu acompanhamento de rotina. A aquisição de imagens foi realizada a 120 kV e 300 mA, com 80 mL de contraste iodado não iônico administrado por via intravenosa a uma taxa de 4 mL/s (iopamidol, 370 mgI/mL). Na apresentação atual, o paciente estava assintomático e seus exames laboratoriais estavam dentro dos limites normais. Microbolhas ao redor da endoprótese foram observadas na janela de tecidos moles (largura da janela: 350 HU, nível: 40 HU) e confirmadas pelo aumento da largura da janela (Figura 1A, seta) em imagens axiais reconstruídas com espessura de corte de 0,625 mm, utilizando reconstrução iterativa híbrida ASiR-V de 50% e um kernel nítido (Bone Plus). As imagens coronais reconstruídas (Figura 1B) e a RHR por TC (Figura 2A e Vídeo 1) não mostraram deformação evidente da endoprótese nem alterações inflamatórias circundantes inicialmente, embora tenha havido um leve aumento na espessura da parede do vaso. A detecção desse novo gás periprotético, ausente em exames de imagem anteriores, gerou preocupação clínica imediata com uma possível infecção do enxerto. O paciente foi internado para observação, conforme orientação médica. No quinto dia de internação, o paciente apresentou febre baixa (temperatura máxima de 37,8 °C) e dor abdominal transitória. A contagem de leucócitos elevou-se para 14,2 × 10^9^/L (valor de referência: 4-10 × 10^9^/L). Uma angioTC de urgência revelou uma massa de tecido mole ao redor da endoprótese, indicando uma coleção inflamatória significativa (Figura 1C). As imagens coronais reconstruídas mostraram a coleção predominantemente na cavidade pélvica (Figura 1D, seta vermelha). Utilizando a RV por TC, medimos com precisão o volume da coleção inflamatória em 698,5 cm^3^, com diâmetros ortogonais máximos de 11,73 cm × 21,70 cm × 11,66 cm no software Advantage Workstation 4.7 (GE Healthcare) (Figura 2B). O paciente foi diagnosticado com infecção por EAA complicada com formação de abscesso secundário e submetido à drenagem percutânea do abscesso guiada por ultrassom. A cultura bacteriana do líquido de drenagem identificou Escherichia coli, sensível à amoxicilina, conforme teste de sensibilidade antimicrobiana. Consequentemente, iniciou-se o tratamento com amoxicilina intravenosa, 1 g a cada 6 horas. Após 7 dias de antibioticoterapia, o quadro clínico do paciente não apresentou melhora. O cirurgião decidiu remover a endoprótese infectada. Durante a cirurgia, constatou-se que a parede da aorta estava íntegra e a massa de tecido mole ao redor da endoprótese não apresentava pulsação. A endoprótese infectada foi removida e a aorta foi reconstruída in situ com um enxerto de Dacron. Infelizmente, porém, o paciente evoluiu para choque refratário intraoperatório e faleceu apesar das tentativas de reanimação.

## Discussão

O EVAR está sendo cada vez mais aplicado no tratamento cirúrgico de AAA. Essa abordagem reduz significativamente o risco de ruptura fatal, mas requer acompanhamento rigoroso a longo prazo^3^ para detectar complicações tardias, como endoleaks, migração do stent e infecção.^4^ Dentre essas, a infecção da EAA é um evento catastrófico com taxa de mortalidade superior a 50% em pacientes sintomáticos.^5,6^ Na maioria dos relatos anteriores, o diagnóstico de infecção era tipicamente estabelecido após o início clínico. No entanto, em nosso caso, suspeitamos da infecção ao detectar gás ectópico via angioTC de corte fino, 5 dias antes do início dos sintomas.^7^ Convencionalmente, a presença de gás ectópico é vista como um sinal de doença avançada, como um abscesso maduro ou fístula. No entanto, nossos achados sugerem que microbolhas identificadas na angioTC de cortes finos podem atuar como um "sinal sentinela" ultraprecoce, aparecendo vários dias antes do início clínico da sepse.^8^

A fisiopatologia da infecção da EAA envolve a colonização bacteriana do biofilme do enxerto, frequentemente originada por contaminação perioperatória ou disseminação hematogênica.^9^ Neste caso, o isolamento de Escherichia coli sugere uma possível origem entérica, possivelmente por translocação bacteriana subclínica ou comunicação microscópica com o trato gastrointestinal.^10^ A fístula entérica foi inicialmente considerada menos provável devido à ausência de sinais clássicos de imagem, como espessamento da parede intestinal ou extravasamento de contraste.^11^ A rápida evolução de microbolhas para uma coleção de 698,5 cm^3^ em cinco dias reflete a alta virulência dos organismos produtores de gás. A detecção precoce da infecção é crucial, pois pode prevenir a disseminação sistêmica e reduzir a sobrecarga geral dos recursos de saúde.^12^ A angioTC é considerada a modalidade de imagem de primeira linha para suspeita de infecção da EAA devido à sua alta sensibilidade para gás ectópico e exsudação periprotética.^13^ Em nosso caso, o uso de aquisição de cortes finos de 0,625 mm combinada com reconstrução iterativa híbrida ASiR-V de 50% foi crucial para distinguir microbolhas periprotéticas de artefatos de imagem. Comparado com exames de acompanhamento de rotina, o aparecimento de novo gás tornou a presença de gás residual pós-operatório (de implante realizado dois anos antes) altamente improvável. Além disso, técnicas avançadas de pós-processamento, como a RV por TC e a RHR por TC, proporcionaram uma compreensão espacial superior da morfologia e das relações entre a prótese e o abscesso.^14^ A integração dessas métricas quantitativas em protocolos de vigilância pode aumentar a concordância interobservador e facilitar o reconhecimento precoce de alterações morfológicas sutis.^15^

**Vídeo 1:** Tomografia computadorizada dinâmica multidirecional com renderização de alta resolução (RHR) demonstrando aneurisma da aorta abdominal, endoprótese e abscesso periprotético. Disponível em: http://abccardiol.org/supplementary-material/2026/12305/2025-0591_IM_Video_1.mp4

O manejo da infecção da EAA continua sendo um desafio devido ao efeito biofilme, no qual as bactérias incorporadas na matriz protética se tornam inacessíveis aos agentes antimicrobianos sistêmicos.^16^ Como observado em nosso caso, mesmo com antibioticoterapia direcionada e drenagem baseada nos resultados da cultura, a infecção se mostrou refratária. Isso ressalta o dilema clínico de equilibrar o tratamento conservador com a explantação cirúrgica radical. A reintervenção cirúrgica em um campo infectado acarreta riscos intraoperatórios extremos,^17^ incluindo hemorragia catastrófica e colapso hemodinâmico, como infelizmente ocorreu neste caso.

## Conclusão

Em conclusão, a detecção de gás ectópico periprotético em angioTC de corte fino serve como um "sinal sentinela" altamente específico de infecção precoce por embolia gasosa aórtica da EAA que pode preceder os sintomas clínicos em vários dias. O reconhecimento desses marcadores subclínicos sutis é crucial para prevenir a progressão para sepse fulminante e suas complicações catastróficas, especialmente considerando a natureza catastrófica da condição e as taxas de mortalidade superiores a 50%. A integração da angioTC de corte fino com técnicas avançadas de renderização é essencial para a identificação precisa. Além disso, como o efeito do biofilme muitas vezes torna a antibioticoterapia isolada insuficiente, os médicos devem manter um baixo limiar para consulta cirúrgica precoce ao detectar sinais progressivos de imagem, mesmo em pacientes assintomáticos.

## Data Availability

Os conteúdos subjacentes ao texto da pesquisa estão contidos no manuscrito.
