# Supplementary material for: Gás Ectópico: Um Sinal de Potencial Infecção de Endoprótese Aórtica Antes do Surgimento de Sintomas
Source: Arq Bras Cardiol. 2026 Jun 16;123(5):e20250591. [Article in Portuguese] doi: 10.36660/abc.20250591 (PMC13399200; doi:10.36660/abc.20250591)
Supplement: Translation

# Translation: Ectopic Gas: A Sign for Potential Aortic Endoprosthesis Infection before Symptoms

**DOI:** 10.36660/abc.20250591i

**Authors:** Hongliang Li, Ying Zhao, Qingyu Ji

**Affiliations:** 1 Baotou Inner Mongolia China Second Affiliated Hospital of Baotou Medical College - Department of radiology, Baotou, Inner Mongolia - China

**Keywords:** Computed Tomography Angiography, Endovascular Aneurysm Repair, Endovascular Procedures, Infections

## Introduction

Abdominal aortic endoprosthesis (AAE) infection is the most dreaded complication of endovascular aortic repair (EVAR) for abdominal aortic aneurysm (AAA), with mortality rates reported as high as 51.8%.^1^ Recent meta-analyses emphasize that despite advances in surgical technique, the prognosis of AAE infection remains poor due to delayed diagnosis and the complexity of endoprosthesis explantation.^1,2^ The management of AAE infection is significantly hindered by diagnostic latency. Currently, computed tomography angiography (CTA) is typically reserved for evaluation only after the onset of infectious symptoms. Although periprosthetic exudation and ectopic gas are considered hallmark radiographic features, these signs are often subtle and minute during the early, pre-symptomatic stages of infection, rendering them easily overlooked. We present a case in which a radiologist detected minute periprosthetic ectopic gas via thin-section CT during a routine CTA follow-up, five days before the onset of infectious symptoms, thereby alerting clinicians to the possibility of AAE infection. Based on this case, we underscore the critical value of thin-section CT in the early identification of ectopic gas. Furthermore, we aim to demonstrate how advanced post-processing—specifically volume rendering (VR) and hyper-realistic rendering (HRR)—enhances the visualization of AAE infection, thereby enabling longitudinal monitoring of infection progression.

## Case presentation

A 78-year-old man, who had a history of EVAR for AAA two years prior, was referred for CTA as part of his routine follow-up. Image acquisition was performed at 120 kV and 300 mA, with 80 mL of non-ionic iodinated contrast administered intravenously at a rate of 4 mL/sec (iopamidol, 370 mgI/mL). At this current presentation, the patient was asymptomatic, and his laboratory values were within normal limits. Microbubbles around the endoprosthesis were observed on soft-tissue window (window width: 350 HU, level: 40 HU) and confirmed by increasing the window width (Figure 1A, arrow) on axial images reconstructed at 0.625-mm slice thickness using 50% ASiR-V hybrid iterative reconstruction and a sharp kernel (Bone Plus). The reconstructed coronal images (Figure 1B) and CT HRR (Figure 2A and Video 1) showed neither evident deformation of the endoprosthesis nor surrounding inflammatory changes initially, although there was a slight increase in vessel wall thickness. The detection of this new periprosthetic gas, which was absent on previous imaging, prompted immediate clinical concern for a potential graft infection. He was admitted for observation according to the doctors’ advice. On day 5 of hospitalization, the patient developed low-grade fever (maximum temperature 37.8 °C) and transient abdominal pain. Leukocyte count rose to 14.2 × 10^9^/L (normal range 4-10 × 10^9^/L). An urgent CTA revealed a soft-tissue mass around the endoprosthesis, indicating a significant inflammatory collection (Figure 1C). The reconstructed coronal images showed the collection predominantly in the pelvic cavity (Figure 1D, red arrow). Utilizing CT VR, we accurately measured the volume of inflammatory collection at 698.5 cm^3^ with maximum orthogonal diameters 11.73 cm × 21.70 cm × 11.66 cm on Advantage Workstation 4.7 (GE Healthcare) (Figure 2B). The patient was diagnosed with an AAE infection complicated with secondary abscess formation and underwent ultrasound-guided percutaneous catheter drainage of the abscess. Bacterial culture of the drainage fluid identified Escherichia coli, which was sensitive to amoxicillin based on antimicrobial susceptibility testing. Accordingly, the patient was started on targeted intravenous amoxicillin 1 g every 6 hours. After 7 days of antibiotic therapy, the patient's clinical condition did not improve. The surgeon decided to remove the infected endoprosthesis. During the surgery, the aortic wall was found to be intact, and the soft-tissue mass around the endoprosthesis had no pulsation. The infected endoprosthesis was removed, and the aorta was reconstructed in situ with a Dacron graft. However, the patient unfortunately developed intraoperative refractory shock and died despite resuscitation efforts.

**Figure 1:** CT angiography (CTA) findings before and after symptom onset. (A) Thin-section axial CTA image (0.625 mm) obtained at routine follow-up reveals ectopic gas (red arrow) around the aortic endoprosthesis. (B) Coronal reconstruction from the same study shows slight thickening of the adjacent vessel wall (arrow). (C) Axial CTA image acquired after symptom onset demonstrates a soft-tissue mass (red arrow), representing an inflammatory collection, around the prosthesis. (D) Coronal reconstruction localizes the resulting periprosthetic abscess (red arrow) within the pelvic cavity.

**Figure 2:** CT hyper-realistic rendering (HRR) before and after symptom onset. (A) At routine follow-up, CT HRR shows no evident deformation of the endoprosthesis or inflammatory changes in the surrounding tissues. (B) After the development of symptoms, CT HRR reveals an extensive inflammatory collection (abscess) around the prosthesis.

## Discussion

EVAR is being increasingly applied in the surgical management of AAA. This approach significantly reduces the risk of fatal rupture but requires rigorous long-term follow-up^3^ to detect late-stage complications such as endoleaks, stent migration, and infection.^4^ Among these, AAE infection is a catastrophic event with a mortality rate exceeding 50% in symptomatic patients.^5,6^ In most previous reports, the diagnosis of infection was typically established after clinical onset. However, in our case, we suspected the infection by detecting ectopic gas via thin-section CTA 5 days before symptom onset.^7^ Conventionally, the presence of ectopic gas is viewed as a sign of advanced disease, such as a mature abscess or fistula. However, our findings suggest that microbubbles identified on thin-section CTA can act as an ultra-early "sentinel sign", appearing several days before the clinical onset of sepsis.^8^

The pathophysiology of AAE infection involves bacterial colonization of the graft's biofilm, often originating from perioperative contamination or hematogenous seeding.^9^ In this case, the isolation of Escherichia coli suggests a potential enteric origin, possibly through subclinical bacterial translocation or a microscopic communication with the gastrointestinal tract.^10^ Enteric fistula was initially considered less probable due to the absence of classic imaging signs such as bowel wall thickening or contrast extravasation.^11^ The rapid evolution from microbubbles to a 698.5 cm^3^ collection within five days reflects the high virulence of gas-producing organisms. Early detection of infection is crucial, as it may prevent systemic spread and reduce the overall burden on healthcare resources.^12^ CTA is considered the first-line imaging modality for suspected AAE infection due to its high sensitivity for ectopic gas and periprosthetic exudation.^13^ In our case, the use of 0.625-mm thin-section acquisition combined with 50% ASiR-V hybrid iterative reconstruction was critical in distinguishing periprosthetic microbubbles from image artifacts. Compared with routine follow-up scans, the appearance of new gas rendered residual postoperative gas (from implantation two years prior) highly improbable. Furthermore, advanced post-processing techniques like CT VR and CT HRR provided a superior spatial understanding of the morphology and relationships between the prosthesis and the abscess.^14^ Integrating these quantitative metrics into surveillance protocols can increase inter-observer agreement and facilitate earlier recognition of subtle morphologic changes.^15^

The management of AAE infection remains challenging due to the biofilm effect, wherein bacteria embedded in the prosthetic matrix are inaccessible to systemic antimicrobial agents.^16^ As seen in our case, even with targeted antibiotic therapy and drainage based on culture results, the infection proved refractory. This underscores the clinical dilemma of balancing conservative management against radical surgical explantation. Surgical re-intervention in an infected field carries extreme intraoperative risks,^17^ including catastrophic hemorrhage and hemodynamic collapse, as unfortunately experienced in this case.

**Video 1:** Dynamic multi-directional CT HRR demonstrating the abdominal aortic aneurysm, endoprosthesis, and periprosthetic abscess. In: http://abccardiol.org/supplementary-material/2026/12305/2025-0591_IM_Video_1.mp4

## Conclusion

In conclusion, new periprosthetic ectopic gas detected on thin-section CTA serves as a highly specific "sentinel sign" of early AAE infection that can precede clinical symptoms by several days. Recognizing these subtle subclinical markers is critical to potentially preventing the progression to fulminant sepsis and its catastrophic complications, particularly given the catastrophic nature of the condition and mortality rates exceeding 50%. Integration of thin-section CTA with advanced rendering techniques is essential for accurate identification. Furthermore, because the biofilm effect often renders antibiotic therapy alone insufficient, clinicians must maintain a low threshold for early surgical consultation upon detecting progressive imaging signs, even in asymptomatic patients.

## Data Availability

The underlying content of the research text is contained within the manuscript.
